# Epigenetic Activation of a Subset of mRNAs by eIF4E Explains Its Effects on Cell Proliferation

**DOI:** 10.1371/journal.pone.0000242

**Published:** 2007-02-21

**Authors:** Yaël Mamane, Emmanuel Petroulakis, Yvan Martineau, Taka-Aki Sato, Ola Larsson, Vinagolu K. Rajasekhar, Nahum Sonenberg

**Affiliations:** 1 Department of Biochemistry, McGill Cancer Centre, McGill University, Montreal, Quebec, Canada; 2 Department of Pathology, College of Physicians and Surgeons, Columbia University, New York, New York, United States of America; 3 Department of Medicine, University of Minnesota, Minneapolis, Minnesota, United States of America; 4 Department of Surgery, Memorial Sloan-Kettering Cancer Center, New York, New York, United States of America; Victor Chang Cardiac Research Institute, Australia

## Abstract

**Background:**

Translation deregulation is an important mechanism that causes aberrant cell growth, proliferation and survival. eIF4E, the mRNA 5′ cap-binding protein, plays a major role in translational control. To understand how eIF4E affects cell proliferation and survival, we studied mRNA targets that are translationally responsive to eIF4E.

**Methodology/Principal Findings:**

Microarray analysis of polysomal mRNA from an eIF4E-inducible NIH 3T3 cell line was performed. Inducible expression of eIF4E resulted in increased translation of defined sets of mRNAs. Many of the mRNAs are novel targets, including those that encode large- and small-subunit ribosomal proteins and cell growth-related factors. In addition, there was augmented translation of mRNAs encoding anti-apoptotic proteins, which conferred resistance to endoplasmic reticulum-mediated apoptosis.

**Conclusions/Significance:**

Our results shed new light on the mechanisms by which eIF4E prevents apoptosis and transforms cells. Downregulation of eIF4E and its downstream targets is a potential therapeutic option for the development of novel anti-cancer drugs.

## Introduction

Post-transcriptional control of gene expression at the level of translation has emerged as an important cellular function in normal development [1] and aberrations in this process leads to diseases including cancer [2], [3]. Translation rates in vertebrates are modulated by a wide variety of extracellular stimuli including hormones, mitogens, growth factors, nutrient availability and stress, and are coupled with cell cycle progression and cell growth [for reviews, see: [4], [5], [6]]. Translation is the most energy-consuming process in the cell [6] and thus, not surprisingly, translation rates are tightly regulated, mainly at the level of initiation [7]. All nuclear-encoded cellular mRNAs possess a cap structure, m^7^GpppN (where N is any nucleotide), at their 5′ terminus [7]. A key player in the regulation of translation initiation is the mRNA 5′ cap–binding protein, eIF4E, which is the limiting component of the eIF4F initiation complex. In addition to eIF4E, this complex contains two other subunits: eIF4A (an ATP-dependent helicase) and eIF4G (a large scaffolding protein), which contains docking sites for the other subunits and additional proteins. eIF4F is directed to the 5′ end of the mRNA via eIF4E and is believed to act through eIF4A (along with eIF4B) to unwind the mRNA 5′ secondary structure to facilitate ribosome binding [7].

Translational control plays an important role in aberrant cell growth and cancer development [reviewed in [8], [9]]. Overexpression of eIF4E results in transformation of immortalized rodent cells [10] and human mammary epithelial cells [11]. eIF4E also cooperates with Myc and E1A to transform rat embryo fibroblasts [12]. Furthermore, antisense- and RNAi-mediated decreases in eIF4E expression results in the inhibition of cell growth and reversion of the transformed phenotype of several cell lines [13]–[16]. eIF4E is also oncogenic *in vivo*, as eIF4E promotes the development of lymphomas and several types of carcinomas in mice [17], [18]. eIF4E protein levels are substantially elevated in many cancers including colon, breast, bladder, lung, prostate, gastrointestinal tract and head and neck cancers; Hodgkin's lymphomas and neuroblastomas [4], [9], [19].

Because eIF4E is the least-abundant initiation factor in most cells [20], [21], it was suggested that mRNAs “compete” for the limiting amounts of eIF4E in the cell [22], [23]. Overexpression of eIF4E results in enhanced translation of mRNAs containing extensive secondary structure in their 5′ untranslated regions (UTRs; [23]). These mRNAs encode proteins such as ornithine decarboxylase (ODC), vascular endothelial growth factor (VEGF) and fibroblast growth factor 2 (FGF2), which are strongly implicated in cell growth, proliferation and survival [reviewed in [9], [19], [24]]. Earlier studies were carried out to systematically identify translation eIF4E targets in cells constitutively overexpressing eIF4E either directly [25], or indirectly through the activation of upstream signaling components that converge on eIF4E [26]. The present study exploits an eIF4E-inducible NIH 3T3 cell line [27], and focuses on very early genome-wide effects of eIF4E overexpression on the recruitment of ribosomes to the mRNAs. This system thus avoids the complexity of secondary effects in established cell lines. Induction of eIF4E expression resulted in increased translation of a group of mRNAs that encode small- and large-subunit ribosomal proteins, anti-apoptotic proteins and cell growth–related factors.

## Results

### Induction of eIF4E in an NIH 3T3 fibroblast cell line

**Figure 1:**
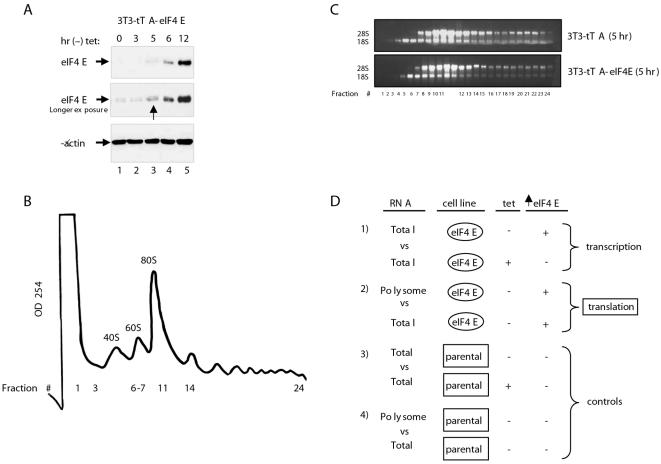
Induction of eIF4E in NIH 3T3 fibroblast cells and microarray analysis. A) eIF4E overexpression was induced by culturing 3T3-tTA-eIF4E cells in a tetracycline free medium. Immunoblots for eIF4E and β-actin were performed. B) A characteristic fractionation profile of 3T3-tTA and 3T3-tTA-eIF4E cells is depicted. Absorbance at 254 nm was monitored. C) Fractions from 3T3-tTA and 3T3-tTA-eIF4E (induced) cells were analyzed on a denaturing agarose gel to visualize the 18S and 28S rRNAs. D) The experimental design used for microarray analysis is shown.

To identify mRNAs whose translation is affected by overexpression of eIF4E, an NIH 3T3 cell line overexpressing eIF4E in a tetracycline-dependent manner was used (3T3-tTA-eIF4E; [27]). eIF4E induction was readily detectable 5 hr after tetracycline removal, and eIF4E levels dramatically increased after 12 hr (Fig. 1A). The eIF4E protein was induced about 3-fold after 5 hr, which is physiologically relevant, as eIF4E amounts can be increased upon cell stimulation and in tumors to a similar extent [28], [29]. To assess the translational effect of eIF4E overexpression and to avoid secondary effects (especially transcriptional), a relatively short induction time of 5 hr was chosen followed by polyribosome fractionation and microarray analysis. Polysome profiles for both induced (-tet 5 hrs) and uninduced 3T3-tTA and 3T3-tTA-eIF4E cells overlapped and showed no significant differences (data not shown). A characteristic profile of 3T3-tTA and 3T3-tTA-eIF4E cells is depicted in Fig. 1B. The 40S and 60S ribosome subunits and 80S ribosomes typically sedimented between fractions 3 and 11 in the sucrose density gradients, whereas polysomes sedimented between fractions 14 and 24 (Fig. 1B). Denaturing agarose gel analysis showed that the 28S rRNA appeared in fractions 6 and 7, where the 60S subunit sedimented (Fig. 1C). Fractions 18–24, which contain the heavier polysomes, were pooled for microarray analysis.

### Microarray analysis of total RNA and polysome fractions

Four sets of microarray experiments were conducted. Two examined the effect of eIF4E induction on transcription and translation in the 3T3-tTA-eIF4E cell line (Fig. 1D, schemes 1 and 2). The other two analyses were conducted using the parental cell line 3T3-tTA which served as a control (Fig. 1D, schemes 3 and 4). First, we examined the effect of eIF4E on total mRNA levels. Total mRNA from uninduced versus induced 3T3-tTA-eIF4E was compared to RNA from uninduced versus induced 3T3-tTA. This analysis identified transcriptional activation that occurs in 3T3-tTA-eIF4E cells when eIF4E is induced but not in 3T3-tTA cells. As predicted from the short induction period for eIF4E, only a limited subset of transcripts was changed in transcriptome abundance (from a total of 27 unique annotated genes, 22 were induced and 5 repressed at a q-value<0.1 and 1.3 fold-change cut off). These changes are probably secondary to translation. As expected eIF4E mRNA levels were increased by approximately 16-fold (Table S1).

Next, we identified the mRNAs that sedimented with the polysomal fractions from induced 3T3-tTA and 3T3-tTA-eIF4E cell lines and normalized them against total RNA (Fig. 1D). This allowed for the scoring of an increase in translational efficiency of a mRNA independent of its abundance in the cell. We then identified those genes whose polysomal/total RNA level differed between 3T3-tTA and 3T3-tTA-eIF4E cells. A preliminary analysis was conducted using a cutoff ratio of 1.5 (Table S2). Further statistical analysis was undertaken (described in Materials & Methods section) and results are shown in Table S3. eIF4E induction resulted in a shift of 294 mRNAs (unique and annotated) to the heavier polysome fractions (Table S3). To search for biological themes that are associated with genes whose translation is affected by eIF4E, we looked for overrepresentation of genes belonging to groups classified by the gene ontology consortium [30]. We separated the dataset into two parts, mRNAs whose translation is activated and mRNAs whose translation is repressed. We considered those that showed a >2-fold relative enrichment and a <0.05 Fisher's test p-value significant. Several functional categories were enriched among genes that were translationally activated including: protein biosynthesis, members of the small and large ribosomal subunits and mitosis related proteins (Table S4).

The recruitment of 245 of mRNAs to ribosomes was decreased (Table S3). The translationally repressed mRNAs were more diverse than the stimulated mRNAs including categories related to differentiation and cell migration (Table S5). While we expected activation of translation as a function of eIF4E induction, the large number of genes whose translation was repressed after eIF4E induction is surprising. Mechanisms that can modulate translational activity of subsets of transcripts exist e.g. micro RNAs (miRNA), which target transcripts for translational silencing [31]–[33]. We hypothesized that miRNA mediated repression, as a possible eIF4E compensatory mechanism, could account for some of the translational repression and searched for overrepresentation of miRNA target sequences among the translationally repressed genes [34]. Interestingly, we found an enrichment of genes with target sites for several miRNAs (14 different miRNAs, Table S5). A similar search among translationally activated transcripts yielded enrichment of only one miRNA (Table S4). This suggests that compensatory miRNA-mediated translational repression might explain a part of the genes that were identified as translationally repressed. Furthermore, untranslated small RNAs/miRNAs have recently been shown to affect the rate-limiting steps of translation initiation [35], [36].

Among the different classes of mRNAs that were recruited to ribosomes, the most unexpected was that ribosomal protein mRNAs from both the small and large subunits (Table S3, Table S4). It is striking, however, that not all ribosomal protein mRNAs were recruited: 26 out of 63 ribosomal protein mRNAs as well as 2 out 13 mitochondrial ribosomal protein mRNAs on the array were recruited to polysomes. Importantly, the sedimentation of growth-related mRNAs was also shifted to heavier polysomes: these include mRNAs encoding anti-apoptotic proteins; growth factors; various cell signaling proteins (kinases, phosphatases); transcription factors and proteins involved in growth and proliferation, mRNA processing, protein degradation and modification, cellular detoxification and transport (Table S3).

### eIF4E overexpression stimulates the translation of a subset of mRNAs

**Figure 2:**
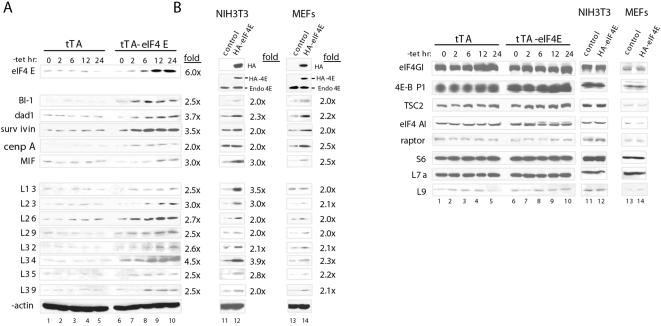
eIF4E induction stimulates the translation of a subset of mRNAs. A) Western blotting of extracts from 3T3-tTA versus 3T3-tTA-eIF4E cells after eIF4E induction (0 to 24 hr) and from NIH 3T3 cells and MEFs that constitutively express HA-eIF4E or vector alone. eIF4E induction was determined by using anti-HA and anti-eIF4E antibodies. Fold increase at the 24 hr time point was determined using NIH Image. B) Western blotting experiments were performed as described in (A). These experiments were repeated three times using three different sets of whole-cell extracts.

To demonstrate that the eIF4E-induced recruitment of mRNAs to polysomes results in enhanced protein levels, several of the proteins of the newly-identified targets were analyzed by Western blotting. The selection of mRNAs was based on the analysis presented above as well as a preliminary analysis of translational activation (Tables S2 and S3). Most genes found in the preliminary analysis were also identified in the final analysis. Some genes failed to pass the more stringent threshold in the final analysis, yet exhibited the translational activation that was predicted, showing that false negatives occur in the present final analysis. To further corroborate our results, NIH 3T3 cells and primary mouse embryonic fibroblasts (MEFs) that constitutively express hemagglutinin-tagged eIF4E (HA-eIF4E) were examined in parallel to the inducible cell line (Fig. 2A and B). HA-eIF4E was detected in the stable cell lines (Fig. 2A, lanes 12 and 14) and was expressed at ∼50% of the level of endogenous eIF4E, assuming that the anti-eIF4E antibody reacts with the HA-tagged protein with an affinity similar to that of eIF4E. We chose to study the levels of several representative eIF4E targets because of the anti-apoptotic and proliferative activities of eIF4E: three anti-apoptotic proteins, bax-inhibitor 1 (BI-1), defender against cell death 1 (dad1) and survivin; the mitosis-related factor centromere autoantigen A (cenpA); the growth- and angiogenic-factor macrophage migration inhibitory factor (MIF) and ribosomal proteins. eIF4E overexpression led to a significant increase (2- to 4-fold) in the amounts of BI-1, dad1, survivin, cenpA and MIF proteins in both the inducible and constitutive cell lines (Fig. 2A). Overexpression of eIF4E also caused a significant increase in the amount of a subset of ribosomal proteins from both ribosomal subunits (Fig. 2A, compare lanes 7–10 to 6). In accordance with these data, increased levels of the ribosomal proteins were also observed in the NIH 3T3 cells and MEFs that constitutively express HA-eIF4E (lanes 12 and 14). To control for specificity, the expression of other translation factors (eIF4GI and eIF4AI), components of the PI3K/Akt/mTOR signaling pathway (TSC2, raptor and the translational repressor 4E-BP1) and β-actin was also determined. No changes in their protein levels were observed in eIF4E-expressing cells (Fig. 2B). Importantly, the levels of ribosomal proteins whose mRNAs did not shift in the polysome gradients (S6, L7a and L9) also failed to increase in response to eIF4E induction (Fig. 2B, lanes 7–10, 12 and 14). These data demonstrate that eIF4E specifically increases the translation of a subset of mRNAs.

**Figure 3:**
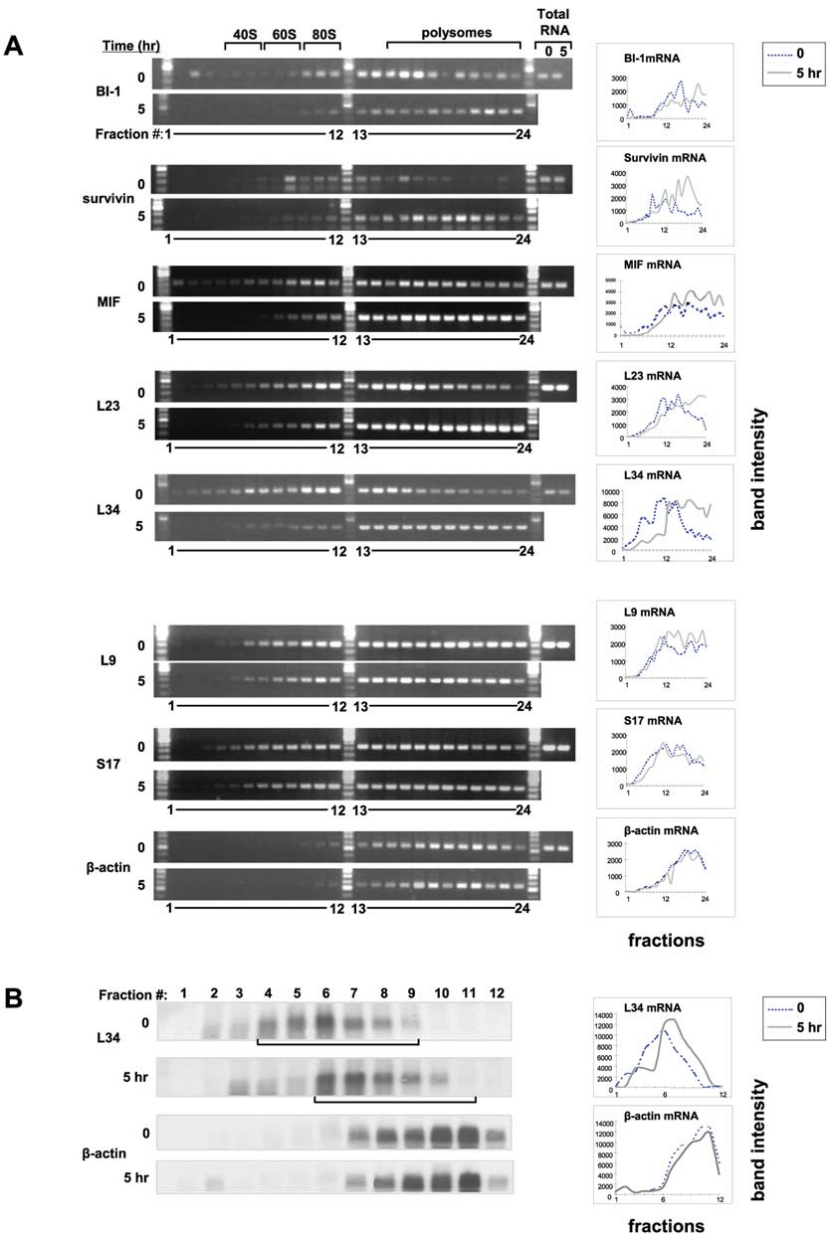
eIF4E induction causes an increase in the recruitment of a subset of mRNAs to polysomes. A) Total and polysomal (24 fractions) RNA from induced (−tet for 5 hr) and uninduced (+tet for 5 hr) 3T3-tTA-eIF4E cells was reverse transcribed into cDNA. Primers for BI-1, survivin, MIF, L23, L34, L9, S17 and actin were used to assess mRNA levels. Amplified PCR bands from the polysomal fractions were quantified using NIH Image, and absolute values were plotted. B) The effect of eIF4E induction on L34 mRNA distribution was assessed by northern blotting. Polysomal RNA was isolated from induced (−tet for 5 hr) and uninduced (+tet for 5 hr) 3T3-tTA-eIF4E cells and fractionated into 12 fractions (for purpose of detection). The RNA was loaded on an agarose denaturing gel and transferred to a nitrocellulose membrane. Membranes were probed with radiolabeled murine L34 and actin probes. Bands were quantified using NIH Image, and absolute values were plotted.

eIF4E induction could in principle either facilitate the recruitment of mRNA to ribosomes or affect protein stability. To distinguish between these two possibilities, RT-PCR was performed on total RNA and polysomal fractions from uninduced and induced 3T3-tTA-eIF4E cells. mRNA levels of two anti-apoptotic proteins (BI-1, survivin), the growth-related factor MIF, a subset of ribosomal proteins (L23, L34, L9, S17) and β-actin were examined. No changes in total mRNA levels were detected after induction (Fig. 3A, last two lanes). Next, the mRNA distribution along the sucrose density gradient was studied. The mRNAs for BI-1, survivin and MIF all shifted to the heavier polysome fractions in the eIF4E-overexpressing cells (Fig. 3A). eIF4E induction also led to increased association of the ribosomal protein mRNAs L23 and L34 with the heavier polysome fractions (Fig. 3A). Two other ribosomal protein mRNAs (L9 and S17) failed to shift to heavier polysomes after eIF4E induction (Fig. 3A). We did not observe changes in β-actin mRNA distribution along the gradient between induced and uninduced cells (Fig. 3A). To ensure that the RT-PCR assay is a valid assay for measuring mRNA distribution along the sucrose density gradient, L34 and β-actin mRNA distribution was also determined by Northern blotting. In agreement with the RT-PCR data, the L34 mRNA shifted to heavier polysome fractions in the eIF4E-overexpressing cells (Fig. 3B). No change in β-actin mRNA distribution was observed (Fig. 3B). Thus, eIF4E overexpression facilitates the recruitment of a subset of mRNAs to polysomes.

### eIF4E knockdown leads to decreased expression of eIF4E target proteins

**Figure 4:**
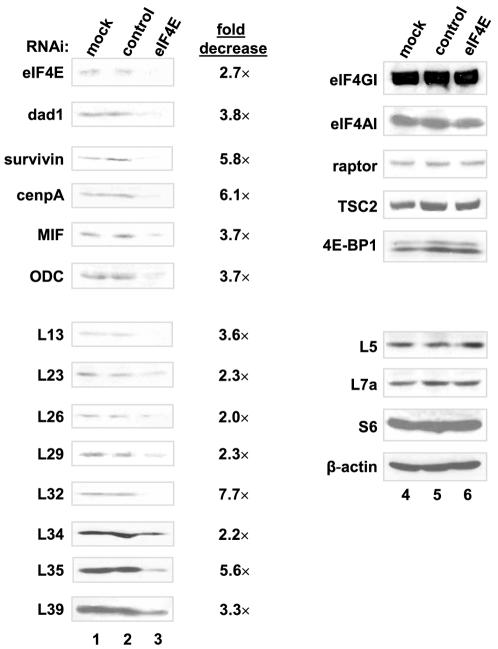
siRNA mediated knockdown of eIF4E in NIH 3T3 cells. NIH 3T3 cells were transiently transfected with an siRNA against murine eIF4E or with a control siRNA, 4E-T-inv (scrambled sequence of human 4E-T), for 48 hr. Cells were lysed, and protein extracts were subjected to SDS-PAGE, followed by western blot analysis. The RNAi-mediated knockdown was repeated three times.

To further ensure that the above described effects are a direct consequence of eIF4E induction, we used small interfering RNA (siRNA)-mediated knockdown of eIF4E to show that the novel targets were affected by eIF4E depletion. An siRNA against murine eIF4E was transiently transfected into NIH 3T3 cells. Another siRNA, 4E-T-inv, which corresponds to a scrambled sequence of the human 4E-transporter protein (4E-T; [37]) and which has no homology to any murine EST or cDNA, was used as a control. Transient transfection of the eIF4E siRNA resulted in a decrease (2- to 3-fold) in eIF4E protein levels relative to the mock-transfected and to the 4E-T-inv siRNA–transfected cells (Fig. 4, compare lanes 1 and 2 to 3). eIF4E knockdown caused a significant decrease (∼4 to 6-fold) in the levels of dad1, survivin, cenpA and MIF protein, which is consistent with our finding that eIF4E overexpression causes their increased expression (compare lanes 1 and 2 to lane 3). The level of ODC, a known target of eIF4E [38], [39], was also significantly reduced (∼4-fold) in eIF4E siRNA transfected cells (Fig. 4). Knockdown of eIF4E also resulted in a decrease of L13, L23, L26, L29, L32, L34, L35 and L39 protein levels (2- to ∼8-fold; Fig. 4). As expected, no changes in the expression of eIF4GI, eIF4AI, raptor, TSC2, 4E-BP1 and β-actin proteins were observed (Fig. 4, lanes 4–6). Furthermore, no decrease in L5, L7a or S6 proteins was seen in these cells (Fig. 4, lanes 4–6). These results confirm that eIF4E preferentially stimulates the translation of a subset of mRNA targets. Our results are summarized in Table S6.

### eIF4E and apoptosis

**Figure 5:**
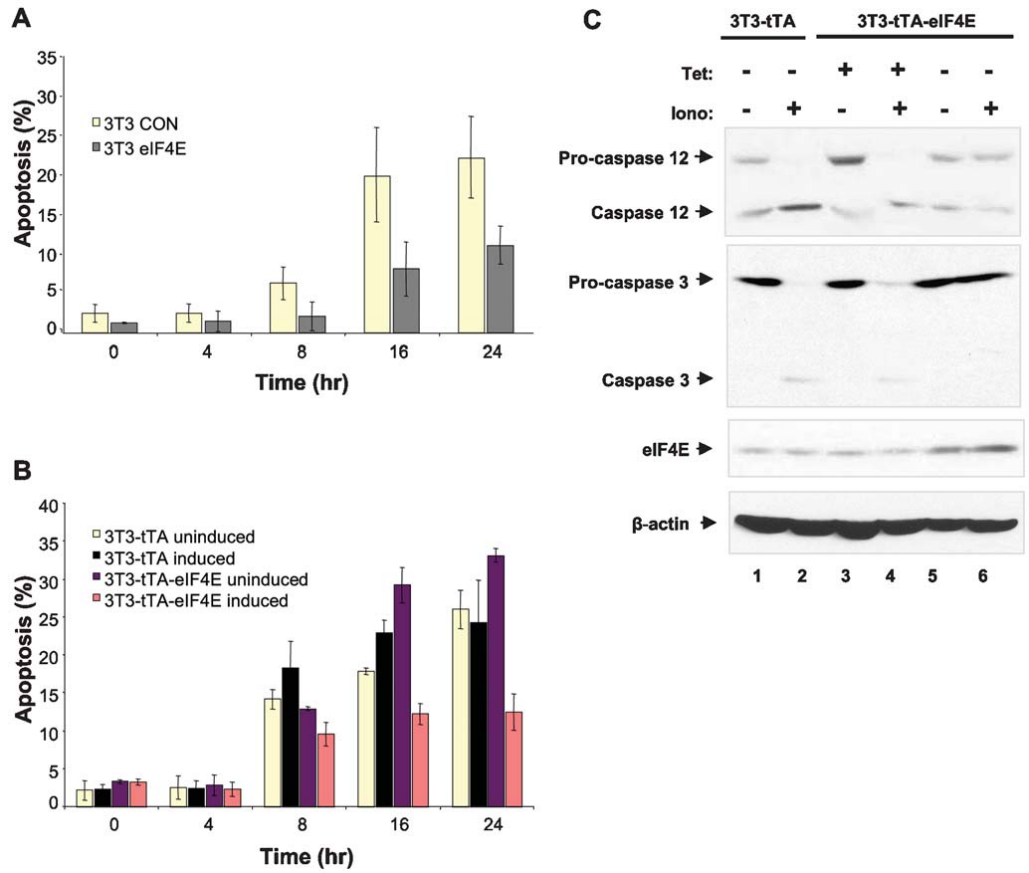
eIF4E induction protects cells against ER-mediated apoptosis. A) 3T3 cells that stably express HA-eIF4E were treated with 5 µM ionomycin for different periods, fixed and stained with propidium iodide. The percentage of apoptosis was quantified by flow cytometry (triplicates were pooled to generate the s.d.) B) 3T3-tTA and 3T3-tTA-eIF4E cells were seeded at 75% confluency and cultured for 8 hr. Tetracycline containing medium was then replaced by a tetracycline free medium for 16 hr to induce eIF4E expression. Uninduced cells were cultured for the same period without removal of tetracycline. Cells were treated with ionomycin and processed as described in (A). C) 3T3-tTA and 3T3-tTA-eIF4E cells were seeded and cultured with or without tetracycline as described in (B). Cells were then cultured for 24 hr in complete medium±5 µM ionomycin. Protein extracts were resolved by SDS-PAGE and transferred to membranes, which were immunoblotted with anti–caspase 3 and anti–caspase 12. eIF4E expression was also examined by immunoblot; elevated eIF4E levels were only detected in 3T3-tTA-eIF4E induced cells (−tet).

eIF4E protects cells against apoptosis [40]. The identification of novel anti-apoptotic eIF4E targets such as BI-1 [41], dad1 [42] and survivin [43] could explain the anti-apoptotic activity of eIF4E. It was therefore pertinent to show that induction of eIF4E protects cells against apoptosis. As previously shown [40], constitutive expression of HA-eIF4E protected cells against ionomycin-induced apoptosis (24 hr: 23% vs. 11% apoptotic; Fig. 5A). The protective effect of eIF4E was even more striking in the eIF4E-inducible cell line because it expresses more eIF4E than does the constitutively expressing HA-eIF4E cell line: eIF4E overexpression in the induced 3T3-tTA-eIF4E cells resulted in a substantial decrease in ionomycin-induced apoptosis (24 hr: 33% vs. 12%; Fig. 5B). No difference was detected in the 3T3-tTA parental cell line (Fig. 5B). eIF4E overexpression also prevented the activation of pro-caspase 12 and pro-caspase 3. When 3T3-tTA and 3T3-tTA-eIF4E cells were induced for 16 hr and then treated with ionomycin, pro-caspase 12 and pro-caspase 3 cleavage to generate their active forms was not observed in induced 3T3-tTA-eIF4E cells. In comparison, pro-caspase cleavage was easily detected in induced 3T3-tTA and uninduced 3T3-tTA-eIF4E cells (Fig. 5C, compare lane 6 to lanes 2 and 4, respectively).

### eIF4E and ribosomal protein mRNA translation

The mechanism by which only a subset of mRNAs are selectively translated upon over expression of eIF4E is not immediately clear. The simplest mechanism would be that features in the UTRs determine which transcripts are selected for translational activation when eIF4E is increased [23]. To test whether any known UTR elements were enriched among genes that were translationally activated, we used the annotation provided by the UTRsite [44]. All elements that showed a >2-fold enrichment and a Fisher's test p-value<0.05 were considered significant. Two known elements were enriched among transcripts that were translationally activated: 5′-TOP (5′-terminal oligopyrimidine) and Mos-PRE (Mos polyadenylation response element) (Table S4).

**Figure 6:**
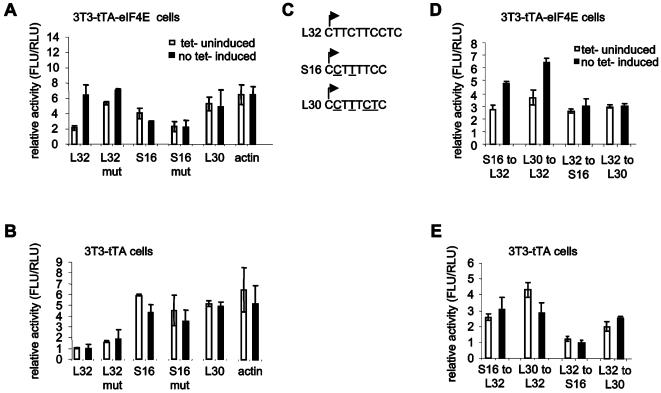
eIF4E and TOP mRNA translation. A) DNA segments encompassing the promoters and 5′ UTRs of L32, L32 mut (non-TOP), S16, S16 mut (non-TOP), L30 and β-actin were subcloned upstream of the firefly luciferase gene. 3T3-tTA-eIF4E cells were transfected with the various firefly luciferase reporters and a renilla luciferase reporter, which was used for transfection efficiency, and were cultured for 32 hr. Tetracycline containing medium was then replaced by a tetracycline free medium for 16 hr; control (non-induced) cells were cultured in parallel with tetracycline. Firefly luciferase activity (FLU) was measured and normalized against renilla luciferase activity (RLU). B) Luciferase activity of the reporters was measured in the parental cell line 3T3-tTA as described in (A). C) TOP sequences of L32, S16 and L30 are depicted. The arrows indicate the transcriptional start site. The nucleotide changes between L32 and S16 and L32 and L30 are underlined. D) Mutated reporters were generated by exchanging the TOP sequences of L32, S16 and L30. Luciferase assays were performed as described in (A). E) Luciferase activity of the reporters was measured in the parental cell line 3T3-tTA as described in (A). Assays were carried out in triplicate. Luciferase activities represent an average obtained from three independent experiments.

The TOP sequence is present at the 5′ end of all mammalian ribosomal protein mRNAs and several translation factor mRNAs, and plays a critical role in their translational regulation [reviewed in 45,46]. An interesting possibility is that only a subset of TOP sequences confers differential eIF4E responsiveness. We therefore examined the effect of eIF4E induction on a TOP-responsive luciferase reporter: DNA segments containing the promoters and 5′ UTRs of murine ribosomal proteins L32, L32 mut (non-TOP), S16, S16 mut (non-TOP), L30 and β-actin (non-TOP) were subcloned upstream of the luciferase gene. Only the L32 ribosomal protein mRNA is an eIF4E target. 3T3-tTA and 3T3-tTA-eIF4E cells were transfected with the various luciferase reporters and induced for 16 hr. Induction of eIF4E led to increased activity of only the TOP-containing L32 5′ UTR reporter (2- to 3-fold; Fig. 6A). Strikingly, mutating the TOP sequence in the L32 5′ UTR abrogated its responsiveness to eIF4E (Fig 6A). eIF4E overexpression did not affect the activity of the mRNAs which contain sequences that do not respond to eIF4E: L30, S16, S16 non-TOP mutant or the β-actin 5′ UTR–containing reporters (Fig. 6A). Luciferase activity of the reporters was also investigated in the parental cell line 3T3-tTA. No increase in activity was detected in these cells (Fig. 6B).

Next, we wished to determine whether the L32 TOP element alone could confer responsiveness to eIF4E independently of the downstream 5′UTR sequence. To this end, the S16 and L30 TOPs were exchanged for the L32 TOP (Fig. 6C). Significantly, eIF4E induction led to increased luciferase activity (2- to 3-fold) of both the mutated S16 and L30 5′ UTRs, which now possessed the L32 TOP sequence (Fig. 6D). The TOP sequence of S16 and L30 was also introduced into the L32 5′ UTR (Fig. 6C). Consistent with the earlier results, the exchange of the L32 TOP for that of S16 and L30 (L32 to S16 and L32 to L30) rendered these mRNAs unresponsive to eIF4E (Fig. 6D). Luciferase activity was also investigated in the parental cell line 3T3-tTA. No increased activity was observed in these cells (Fig. 6E). Taken together, these results clearly demonstrate that eIF4E overexpression affects only a subset of TOP-containing mRNAs.

## Discussion

eIF4E is thought to stimulate the translation of a subset of mRNAs whose 5′ UTRs contain extensive secondary structure [23]. A prevailing model postulates that eIF4E functions as part of the eIF4F complex to melt the 5′ mRNA secondary structure to facilitate ribosome binding and scanning [7]. Because eIF4E is the limiting factor for eIF4F assembly, it determines the availability of eIF4F for unwinding of mRNA secondary structures [20], [21]. Through the use of microarray analysis of polysome fractions, we identified novel eIF4E targets that are involved in ribosome biogenesis, cell proliferation and survival. Many of these mRNAs are predicted to contain extensive secondary structure (e.g., BI-1, dad1, survivin, cenpA and MIF). We also identified mRNA targets that are not predicted to contain extensive 5′ secondary structure, partly because the search for targets was biased against such mRNAs. Two earlier studies suggested that eIF4E could stimulate ribosomal protein mRNA translation both *in vivo* and *in vitro* [47], [48]. Our study shows the importance of eIF4E in the translational regulation of a subset of ribosomal protein mRNAs.

A large body of evidence is consistent with the notion that synthesis of ribosomal proteins is required for cell growth, proliferation and survival [46], [49]–[51]. Increased ribosomal protein and rRNA synthesis promote the assembly of ribosomes and subsequently affect the rate of protein synthesis. Many types of cancers exhibit elevated amounts of ribosomal proteins [49], [52]–[55]. They also often exhibit higher rates of protein synthesis activity that are proportionate to their increased growth and proliferation [49].

Several of the ribosomal proteins identified here as targets of eIF4E are upregulated in different cancers. For example, S13 and L23 are upregulated in multi-drug-resistant gastric cancers and promote multi-drug resistance by suppressing apoptosis [56]. L32 expression correlates with the progression of human prostate cancer [57], and S27 is overexpressed in melanomas [58]. In this report, we show that eIF4E overexpression affects the translation of only a subset of TOP mRNAs. Thus, one possibility is that some of the functions of ribosomal proteins in control of cell growth, proliferation, differentiation and survival are extra-ribosomal [49], [59]. This is consistent with a lack of increase in rRNA levels or differential rRNA processing in the eIF4E-overexpressing cells (unpublished observations).

Rapamycin inhibits mTOR activity and consequently diminishes eIF4E function [60]. Grolleau et al. identified mRNAs whose translation is suppressed by rapamycin treatment of Jurkat T cells [61]. Many of the mRNAs whose association with polysomes was decreased by rapamycin were also identified here, such as survivin, dad1, ribosomal proteins S7, S9, S13, S21, S24, S26, S27, L13, L23, L29, L32, L34 and L35, proteasome subunit β type 1 and type 3, protein tyrosine phosphatase 4A2, cyclophilin A, glutathione-*S*-transferase and the ATPase synthase subunit C. Another study has also shown that TOR inhibition in yeast leads to a significant decrease in the translation of mRNAs encoding initiation factors and ribosomal proteins [62]. These results are important in establishing a direct link between the tumorigenic properties of components of the PI3K/Akt/mTOR pathway and translation initiation via eIF4E. mTOR phosphorylates directly or indirectly several translation factors including 4E-BPs, S6Ks, eIF4G, eIF4B and eEF2 [reviewed in 63,64,65]. Because inhibition of mTOR by rapamycin results in a reduction of similar set of mRNAs whose translation is also stimulated by eIF4E [61], it can be concluded that mTOR stimulates translation via its phosphorylation of the eIF4E repressors, the 4E-BPs. Thus, eIF4E is an important target of the PI3K/Akt/mTOR pathway that mediates its wide-ranging cellular activities [63], [65]–[67].

A recent study that was published after this paper was completed also identified targets of eIF4E [25]. However, this study used stably eIF4E-expressing NIH 3T3 cell line. Thus, it is likely that some of the results published by Larsson et al. are due to secondary effects of eIF4E overexpression. We used a relaxed significance threshold (q<20%, as defined in SAM) as previously described [25], to identify genes that are differentially translated in each study and then identified the overlap. This approach resulted in identification of 340 cDNA clones representing 214 unique genes with gene identifiers. The list included several ribosomal proteins. The lack of a larger overlap is most likely due to a combination of biological and technical issues. The biological differences include the model system, a short period of eIF4E induction in the present study and the possible secondary consequences of constant eIF4E expression in the previous report. Technical differences include the definition of the translationally active pool, study design, differences in protocols used for microarray labeling and hybridization and usage of a cDNA platform that has been shown to show higher variability in a high proportion of cases (although it is beyond the scope of this study to define the performance of the platform across labs, chip-versions and batches) [68].

This study identified novel anti-apoptotic eIF4E targets, including survivin, which has a well-documented role in cancer cell growth and cell survival [43]. Survivin is highly expressed in several cancers, and its expression is sensitive to PI3K/Akt/mTOR inhibitors [69], [70]. CenpA, dad1, BI-1 and MIF are also overexpressed in human cancers [71]–[75]. eIF4E-mediated expression of these proteins would therefore significantly increase cellular proliferation and inhibit apoptosis. In summary, overexpression of eIF4E preferentially stimulates the translation of a subset of mRNAs that encode small- and large-subunit ribosomal proteins, anti-apoptotic proteins and cell growth–related factors. Deciphering how the translational machinery preferentially translates these mRNAs will lead to a better understanding of the proteome's regulation and its involvement in diseases such as cancer.

## Materials and Methods

### Generation of eIF4E-overexpressing cell lines

The eIF4E-inducible NIH 3T3 (3T3-tTA-eIF4E- TET OFF system) cell line was previously described [27]. The 3T3-tTA parental cell line was used as control. The cell line that stably overexpresses eIF4E was generated using the pBabe retroviral system (Clontech). The packaging cell line Phoenix Ecotropic (transformed human embryonic kidney cells) was cultured in DMEM with 10% FBS. The packaging cell line was transfected as described by Dr. G. Nolan's protocols (www.stanford.edu/group/nolan/phx_helper_free.html) with two vectors: pBabewt (no insert) and pBabe-HA-eIF4E (3 HA tags). Virus was harvested from the packaging cell line 48 hr after transfection and was used to infect NIH 3T3 cells and MEFs. Cells were infected twice (every 24 hr) in the presence of 8 µg/ml polybrene (Sigma). Transduced NIH 3T3 cells were subjected to drug selection (2 µg/ml puromycin, final concentration) 48 hr after infection. A polyclonal population resistant to puromycin was used for analysis of protein expression. Primary MEFs (passage 4) were used to generate the HA-eIF4E-expressing MEFs as described above. Selection of MEFs was performed for 3 days. A polyclonal population resistant to puromycin was used for analysis of protein expression. Cells were lysed in 40 mM HEPES-KOH [pH 7.5], 120 mM NaCl, 1 mM EDTA, 10 mM pyrophosphate, 10 mM glycerophosphate, 50 mM NaF, 1.5 mM Na_3_VO_4_, 0.3% CHAPS and one tablet EDTA-free protease inhibitors (Roche) for 10 min on ice. Protein supernatants were recovered after a 10-min spin at 14,000 g. Protein concentration was determined using the Bradford reagent (BioRad). Protein extracts were subjected to SDS-PAGE and transferred to a nitrocellulose membrane, which was probed with various antibodies as described below.

### Sucrose gradient fractionation and polysome isolation

3T3-tTA-eIF4E and 3T3-tTA cells were grown in 150-mm dishes to 80% confluency. eIF4E expression was induced by removing tetracycline from the medium for 5 hr; cells were then treated with cycloheximide (100 µg/ml) for 15 min at 37°C. Cells were washed three times in cold PBS containing 100 µg/ml cycloheximide and were scraped off the plate using a rubber policeman and 1 ml of the same solution. Cells were centrifuged for 5 min at 1,000 rpm and resuspended in 850 µl hypotonic lysis buffer (5 mM Tris-HCl, pH7.5; 2.5 mM MgCl_2_; 1.5 mM KCl). Cells were transferred to a pre-chilled tube and incubated with 100 µg/ml cycloheximide, 2 mM DTT and 2 µl RNAsin Inhibitor (40 U/µl; Stratagene). Cells were incubated on ice for 5 min and vortexed. To each 850 µl of cells, 50 µl of 10% Triton X-100 and 50 µl of 10% sodium deoxycholate were added; cells were then vortexed and incubated on ice for 5 min. Cell extracts were centrifuged for 5 min at 14,000 rpm; the supernatants were collected and loaded onto a pre-chilled 10–50% sucrose gradient. Each gradient was formed by mixing 5.5 ml of 10% and 50% sucrose in a Beckman Centrifuge tube (14×89 mm; Beckman Instruments #3311372, CA, USA) using a Labconco pump (Kansas City, MO, USA). Gradients were placed in a Beckman SW40Ti rotor and centrifuged at 35,000 rpm for 2 hr at 4°C. Fractions were collected (24 fractions of 12 drops each) using a Foxy JR ISCO collector and UV optical unit type 11 (St-Lincoln, NE, USA).

### RNA isolation for microarrays

Total RNA was isolated using Trizol as described by the manufacturer (Invitrogen). RNA from sucrose gradients was isolated by using Trizol at a 1∶1 ratio (500 µl of fraction and 500 µl of Trizol). The last seven fractions (#18–24; heavy polysomes) were pooled, extracted with phenol-chloroform, ethanol precipitated and used for microarray analysis. RNA purity and integrity were assessed by spectrophotometric analysis (OD 260/280) and denaturing agarose gel.

### Microarray analysis

Total or polysomal RNA (10 µg) was used for microarrays. Microarrays were performed using a direct labeling protocol according to the UHN Microarrray Centre (Toronto, Canada, http://www.microarrays.ca/support/proto.html). Mouse 15K3 cDNA arrays were purchased from the UHN Microarrray Centre; they were scanned and the resulting images were digitalized using Axon GenePix 4000B scanner and software. Preliminary analysis was conducted using Iobion software (lowess approach) with a cutoff ratio of 1.5. The data from the final analysis was normalized using the bioconductor [76] library “limma” [77] and the non-print tip lowess approach without background subtraction [78] as this increases the accuracy of the obtained measurements [68]. Replicate spots on each array were separated and treated as independent. After normalization we identified structural variation from both dye bias and experimental set using principal components analysis (PCA, data not shown). We therefore used a paired analysis where samples were stratified based on dye and experimental set. This pairing did not introduce any bias in the analysis regarding these sources of structural variation. After pairing the biological theme, where the estimates from translational efficiency from two cell lines could be separated, was apparent in PCA components describing a large proportion of the total data set variation (data not shown). This indicates that the data analysis approach is valid.

Significant differentially expressed/translated genes were identified using the Significance Analysis of Microarrays (SAM) algorithm using a q-value threshold of q<0.1 [79]. SAM was performed in R (r-project.org) using the library (“samr”) and a “Two class paired” analysis. No data filtering was performed before SAM as described [80]. mRNAs whose polysomal/total were changed by at least 1.3-fold (q-value<0.1) between the eIF4E-overexpressing cell line and the parental cell line were considered significant. As we initially identified an over-representation of ribosomal genes and these tend to be highly expressed, which reduces the dynamic range of their fold changes, we used a relatively modest fold change criteria (1.3) in combination to our statistical threshold (q<0.1). The dataset has been submitted to GEO accession number GSE6639, including raw data files to enable future integrative analysis [81].

We used the Perl module GO: Termfinder [82] to assess overrepresentation of functional terms as defined by the gene ontology consortium [83], known RNA elements [44] and miRNAs [34], targetscan v3.0) among genes that were identified to be translationally regulated. The analysis was stratified into two parts assessing overrepresentation among genes that were translationally activated and translationally repressed. For this analysis, no fold change filtering was performed.

### Experimental design

All microarray comparisons were performed in quadruplicates using dye swaps performed on the same RNA extraction (thus 4 arrays were used per sample class derived from two separate RNA extractions).

For changes at the transcriptional level, total RNAs from the 3T3-tTA and 3T3-tTA-eIF4E cell lines were compared. Microarray experiments were performed on total RNA from uninduced (+tet for 5 hr) versus induced (−tet for 5 hr) 3T3-tTA cells, and the obtained ratios were compared to the ratios of total RNA from uninduced (+tet for 5 hr) versus induced (−tet for 5 hr) 3T3-tTA-eIF4E cells using the paired analysis described above.

For changes at the level of translation, total RNA was compared to heavy polysomal RNA from both 3T3-tTA and 3T3-tTA-eIF4E induced (−tet 5 hr) cell lines. Changes in the ratio between polysomal vs total RNA levels between 3T3-tTA and 3T3-tTA-eIF4E induced cells were identified using the paired analysis described above.

### Western blot analysis

Whole-cell extracts (NIH 3T3, 35–50 µg protein; MEFs, 125–250 µg) were resolved on a 4–20% gradient SDS-PAGE and transferred to a nitrocellulose membrane (BioRad). The membrane was blocked in 5% milk/1× PBS for 1 hr and probed overnight at 4°C with the appropriate antibody. The signal was detected with secondary antibodies conjugated to horseradish peroxidase at a dilution of 1∶1,000 and developed with chemiluminescence substrate (Amersham). Antibodies were purchased or received from the following sources: α-eIF4E (BD Biosciences); α-dad1 (A. Winoto, University of California, Berkley, CA, USA); α-cenpA (W. Lee, Murdoch Children's Research Institute, Parkville Victoria, Australia); α-MIF (T. Roger, Infectious Diseases Service CHUV, Lausanne, Switzerland); α-Bax-inhibitor-1 (MBL International); α-survivin (Novus Biologicals); α-L13, -L29, -L32, -L34, -L35 and -L39 (described previously in [84]; α-L26 (M. Kastan, St. Jude Children's Research Hospital, Memphis, TN, USA); α-L5 and α-L23 (H. Lu, Oregon Health & Sciences University, OR, USA.); α-L7a (S. Fumagalli, University of Cincinnati, OH, USA); α-L9 (Transduction Laboratories); α-eIF4GI N-terminal [85]; α-eIF4AI [86]; α-raptor (unpublished data); α-S6 and α-4E-BP1 (Cell Signaling Technology); α-TSC2 (Santa Cruz) and α-actin (Sigma).

### RT-PCR assays

RT-PCR was performed with the BD Biosciences RT-PCR kit (K1402-2). For the reverse transcription reaction, 1 µg of total RNA and 1 µl of each polysome fraction were used. The diluted cDNA (2 µl from a 1 in 100 dilution) was used in a 20 µl PCR reaction with Precision TaqPlus (Stratagene). Aliquots were loaded on a 1.5% agarose gel and visualized by ethidium bromide staining and UV shadowing. A 540 bp actin fragment was amplified using the mouse β-actin control amplimer set (BD Biosciences, #5408-1). All other cDNAs were amplified by PCR primers purchased from Invitrogen (all noted 5′-3′): BI-1-5′, GCCAGTTTTGCACTATGTATG; BI-1-3′, AGAGAGGACAGGAGCATGAG; Survivin 5′, CAGATCTGGCAGCTGTACCT; Survivin 3′, GGCTCTCTGTCTGTCCAGTT; MIF-5′, CTTATGTTCATCGTGAACACC; MIF-3′, GCGTTCATGTCGTAATAGTTGA; L23-5′, AGGACGCGGTGGGTCCTC; L23-3′, GCGTTGGATGCAATTCTGGG; L34-5′, ATGGTCCAGCGTTTGACATAC; L34-3′, ACTCTGTGCTTGTGCCTTCAA; L9-5′, AACATGATCAAGGGTGTCACG; L9-3′, GATGCCGTCCAAAAACTTCCT; S17-5′, TCATCGAGAAGTACTACACGC and S17-3′, CTGAGTGACCTGAAGGTTAG. Increasing PCR cycles (18–33) and cDNA from total RNA were used to determine the number of cycles needed for amplification in the linear range for each transcript. The linear range was determined to be 23 cycles for β-actin and 28 cycles for MIF, survivin, L23, L34, L9 and S17. RT-PCR quantification was performed using NIH Image software.

### Northern blot analysis

3T3-tTA-eIF4E cells from five 150-mm plates (70–80% confluency) that were induced by removing tetracycline from the medium (−tet for 5 hr) or that were uninduced (+tet for 5 hr) were collected, lysed and fractionated by sucrose gradient ultracentrifugation (as described above). RNA was fractionated into 12 fractions unlike for RT-PCR assays (24 fractions) for purpose of detection. The fractions were extracted using Trizol (as described above). RNA fractions were heated at 70°C for 10 min in RNA sample buffer, rapidly cooled on ice and loaded on a MOPS-formaldehyde agarose gel (Northern Max Kit, Ambion). RNAs were separated at 85 V, and visualized by UV shadowing. RNA was transferred to a BrightStar Plus membrane (Ambion) and crosslinked to the membrane by UV irradiation. cDNA fragments from L34 and actin were generated by PCR (refer to primers described above for RT-PCR). Denatured cDNAs were labeled using the Ready-to-Go DNA labeling kit (Amersham) in the presence of [α-^32^P]dCTP. Unincorporated dCTP was removed using micro Spin S-200 HR columns (Amersham). Membranes were prehybridized for 30 min at 42°C with hybridization solution (Northern Max Kit). Membranes were then incubated with 1×10^6^ cpm/ml of radiolabeled probe in hybridization solution overnight at 42°C. Membranes were washed as described in the manual (Northern Max Kit) and exposed to film.

### RNAi

siRNA against eIF4E was designed by Dharmacon software. Cells were seeded in 6-well plates at 20% confluency and transfected the next day using Lipofectamine Plus reagent (Invitrogen). On the day of transfection, cells were washed twice and incubated with 750 µl of OptiMem (Invitrogen) per well. The transfection protocol was as follows: in a polystrene tube (for one 6-well plate), 645 µl of OptiMem was added to 45 µl of 40 µM eIF4E or 4E-T inverted control siRNA (Dharmacon) and 60 µl of PLUS reagent (Invitrogen) and this mixture was incubated 15 min at room temperature. A mixture of OptiMem (690 µl) and Lipofectamine reagent (60 µl) (Invitrogen) was added and further incubated for 15 min at room temperature. Each well received 250 µl of the combined mixture, and the plates were incubated for 3 hr at 37°C. The transfection mixture was removed and replaced with complete medium. Cells from each 6-well plate were trypsinized and seeded onto one 150-mm plate 24 hr after transfection. Cells were harvested for western blotting and polysome fractionation 48 hr after transfection.

### Apoptosis assays

NIH3T3 cells that stably overexpress HA-eIF4E were cultured in 100-mm dishes at 75% confluency in complete medium with or without 5 µM of ionomycin (Sigma). The eIF4E-inducible cells, as well as the parental cells, were seeded in 100-mm dishes at 75% confluency and cultured for 8 hr, at which point tetracycline was removed from the medium to induce eIF4E for 16 hr. On the next day, both the inducible and parental cells were treated with 5 µM of ionomycin for 4, 8, 16 or 24 hr. All cells were fixed in 70% ethanol, stained with propidium iodide (50 µg/ml) and treated with RNAse A (20 µg/ml) in 1× PBS/5 mM EDTA for 1 hr at room temperature. The percentage of apoptosis was quantified by flow cytometry. For caspase 3 and caspase 12 immunoblots, cells were seeded in 150-mm dishes at 75% confluency and cultured for 8 hr. Cells were treated with a tetracycline free medium to induce eIF4E for 16 hr, after which the cells were cultured for 24 hr in complete medium with or without 5 µM of ionomycin. Cells were lysed as described above. Protein extracts (100 µg) were subjected to SDS-PAGE and immunoblotted with anti–caspase 3 and anti–caspase 12 antibodies (Cell Signaling Technology).

### Constructs

The plasmids for 5′ UTRs of murine β-actin, L32, L32 mut (non-TOP; C-A mut), S16, S16 mut (non-TOP) and L30 were provided by O. Meyuhas (Hebrew University, Jerusalem, Israel). DNA was PCR-amplified with PWO polymerase (Stratagene) and subcloned into the pGL3 basic-luciferase reporter (Promega). Primer sequences (including restriction enzymes sites, which are underlined) are as follows (all noted 5′-3′): 5′L32 and actin, ACGTACGCGTCGACGGCCAGTGCCAAG; 3′L32, ACGTAGATCTTTGGGATCCG GCAGCCAC; 3′actin, ACGTAGATCTTTGGGATCCTCTAGAGTCG; 5′S16wt and mut, ACGTACGCGTAAGCTTGCATGCACAGCT; 3′S16wt, ACGTCTCGAGGGATCCTCTAGCCACACC; 3′S16mut, ACGTCTCGAGGGATCCCCACACCGCAG; 5′L30wt, ACGTACGCGTAAGCTTCAGAACAAACGCC and 3′L30wt, ACGT CTCGAGGGATCCTCTAGCCAGCCG. Constructs were verified by restriction enzyme digests and were sequenced using two oligonucleotides: sense, CTAGCAAAATAGGCTG TCCCC, and reverse, TTTATGTTTTTGGCGTCTTCC.

The wild-type S16 and L30 TOP sequences were replaced by the TOP sequence of L32. The primers listed below were used to generate the mutated constructs S16 to L32, L30 to L32, L32 to S16 and L32 to L30 (restriction enzyme sites are underlined (all noted 5′-3′): 5′S16-L32, ACGTACGCGTAAGCTTGCATGCACAGCTCCGC; 3′S16-L32, ACGTCTCGAGGGATCCTCTAGCCACACCGCAGCGCCGCGACCGGAAGAAGGAAGAGGGGGCCAACCCAGCCGATTTT; 5′L30-L32, ACGTACGCGTAAGCTTCAGAACAAACGCCCAGA; 3′L30-L32, ACGTCTCGAGGGATCCTCTAGCCAGCCGCCAAGATGGCCGGGGAGCGGAAGAAGGAAGAGGTCCCACAATGCAAAGCTCTTCTA; 5′L32-S16 and -L30, ACGTACGCGTAAGCTTGCATGCCTGCAGGT; 3′L32-S16, ACGTCTCGAGGGATCCGGCAGCCACCTCGTAGGCAGCGCCGAGGAAAAGGGAAGCGCCGGCGGCGGCGCGCAAGG and 3′L32-L30, ACGTCTCGAGGGATCCGGCAGCCACCTCATGGGCAGCGCCGAGAGAAAGGAAGGAGCCGGCGGCGGCGCGCAAGG.

### Luciferase assays

Cells were seeded in 6-well plates (5×10^5^ cells/well), transfected with 2.5 µg of Firefly luciferase reporter and 250 ng of pRL-TK (Renilla luciferase reporter) for transfection efficiency (Promega) and were cultured for 32 hr. Cells were then induced to overexpress eIF4E by removing tetracycline for 16 hr. Transient transfections with Lipofectamine Plus reagent (Invitrogen) were performed according to the manufacturer's protocol. Cells were lysed, and luciferase assays were carried out according to a standard protocol for the dual luciferase assay system (Promega).

## Supporting Information

Table S1 Transcriptionally regulated mRNAs in eIF4E-overexpressing 3T3 cells. Total RNA from eIF4E-induced NIH 3T3-tTA-eIF4E (-tet 5 hr) versus uninduced (+tet 5 hr) cells was compared to 3T3-tTA cells with and without tetracycline. Genes showing fold changes of >1.3 and SAM q<0.1 were considered significant.
(0.02 MB XLS)

Table S2 Preliminary analysis of translationally activated genes. The mRNAs that sedimented with polysomes from induced 3T3-tTA-eIF4E (-tet 5 hr) cells were compared to total RNA from the same cells. The ratios were normalized against those obtained by comparing polysomal RNA of 3T3-tTA (-tet 5 hr) cells versus total RNA from the same cells. Average fold changes of 1.5 and over were considered significant.
(0.11 MB DOC)

Table S3 Translationally regulated mRNAs in eIF4E-overexpressing 3T3 cells. Genes that experience a change in polysomal/total RNA ratio between 3T3-tTA-eIF4E and 3T3-tTA are shown. Fold changes of >1.3 and SAM q-value<0.1 were considered significant.
(0.14 MB XLS)

Table S4 Functional analysis of translationally activated genes. Genes that were translationally activated by induced eIF4E (SAM q<0.1) were searched for overrepresentation of functional categories as defined by the gene ontology consortium, the UTRSite and the targetscan miRNA database.
(0.03 MB XLS)

Table S5 Functional analysis of translationally repressed genes. Genes that were translationally repressed by induced eIF4E (SAM q<0.1) were searched for overrepresentation of functional categories as defined by the gene ontology consortium, the UTRSite and the targetscan miRNA database.
(0.03 MB XLS)

Table S6 Summary table of ribosomal protein mRNA changes induced by eIF4E overexpression. Results from the microarray analyses (preliminary and final), figures 2, 3 and 4 for ribosomal protein mRNAs are summarized.
(0.02 MB XLS)
