# Supplementary material for: Epigenetic Activation of a Subset of mRNAs by eIF4E Explains Its Effects on Cell Proliferation
Source: PLoS One. 2007 Feb 21;2(2):e242. doi: 10.1371/journal.pone.0000242 (PMC1797416; doi:10.1371/journal.pone.0000242)
Supplement: Table S2 — Preliminary analysis of translationally activated genes. The mRNAs that sedimented with polysomes from induced 3T3-tTA-eIF4E (-tet 5 hr) cells were compared to total RNA from the same cells. The ratios were normalized against those obtained by comparing polysomal RNA of 3T3-tTA (-tet 5 hr) cells versus total RNA from the same cells. Average fold changes of 1.5 and over were considered significant. (0.11 MB DOC) [file pone.0000242.s002.doc]

**Table S2. Preliminary analysis of translationally upregulated mRNAs in eIF4E-overexpressing 3T3 cells**

| **GenBank accession #** | **Gene description** | **Average**  **fold change** | **GenBank accession #** | **Gene description** | **Average fold change** |
| --- | --- | --- | --- | --- | --- |
|  |  |  |  |  |  |
|  | **Ribosomal proteins-large subunit** |  |  | **Cell cycle** |  |
| NM_009076 | ribosomal protein L12 | 1.6 | NM_013812 | CDK2 (cyclin-dependent kinase 2)-associated protein 1 | 1.9 |
| NM_016738 | ribosomal protein L13 | 2.5 |  |  |  |
| BC091759 | ribosomal protein L17 | 1.7 |  | **Growth factor/immune response** |  |
| NM_022891 | ribosomal protein L23 | 1.5 | NM_010798 | macrophage migration inhibitory factor | 3.6 |
| NM_009080 | ribosomal protein L26 | 1.6 |  |  |  |
| NM_009082 | ribosomal protein L29 | 1.6 |  | **Cellular detoxification** |  |
| NM_172086 | ribosomal protein L32 | 1.7 | NM_026764 | glutathione-*S*-transferase mu4 | 2.1 |
| NM_026724 | ribosomal protein L34 | 2.5 |  |  |  |
| NM_025592 | ribosomal protein L35 | 2.3 |  | **Protein modification/degradation** |  |
| NM_026055 | ribosomal protein L39 | 1.9 | NM_175190 | similar to serine hydroxymethyltransferase, mitochondrial precursor | 1.8 |
| BF582862 | sequence with strong similarity to human ribosomal protein L37a | 2.1 | NM_011822 | phosphatidylinositol glycan, class Q | 1.7 |
| NM_026759 | mitochondrial ribosomal protein L13 | 1.8 | NM_011185 | proteasome (prosome, macropain) subunit, beta type 1 | 2.0 |
|  |  |  | NM_011184 | proteasome (prosome, macropain) subunit, alpha type 3 | 2.0 |
|  | **Ribosomal proteins-small subunit** |  | NM_008907 | cyclophilin A | 2.4 |
| BC099605 | ribosomal protein S7 | 2.2 | NM_009456 | ubiquitin-conjugating enzyme E2L 3 | 1.7 |
| NM_029767 | ribosomal protein S9 | 2.1 |  |  |  |
| NM_026533 | ribosomal protein S13 | 1.6 |  | **Transport** |  |
| NM_026147 | ribosomal protein S20 | 1.6 | BC098508 | importin 9 | 2.4 |
| NM_025587 | ribosomal protein S21 | 1.6 | NM_207530 | oxysterol binding protein-like 1A | 1.7 |
| NM_011297 | ribosomal protein S24 | 2.1 | NM_010860 | myosin light chain, alkali, nonmuscle | 2.3 |
| NM_024266 | ribosomal protein S25 | 2.1 | NM_016903 | esterase 10 | 2.0 |
| NM_013765 | ribosomal protein S26 | 1.6 | NM_146116 | tubulin beta 2 | 1.9 |
| NM_027015 | ribosomal protein S27 | 1.5 | NM_022813 | secretory carrier membrane protein 2 | 1.7 |
|  |  |  |  |  |  |
|  | **Translation and mRNA processing factors** |  |  | **Membrane or cellular components** |  |
| NM_007917 | eukaryotic translation initiation factor 4E | 1.5 | NM_024214 | translocase of outer mitochondrial membrane 20 homolog | 1.7 |
| NM_011508 | suppressor of initiator codon mutations, related sequence 1 | 1.8 | NM_011512 | surfeit gene 4 | 1.8 |
| NM_025840 | basic leucine zipper and W2 domains 2 | 1.8 | NM_019435 | nuclear protein 15.6 | 1.7 |
| NM_026030 | eukaryotic translation initiation factor 2, subunit 2 (beta) | 1.9 | NM_025849 | RIKEN cDNA 3110001D03 gene | 1.8 |
| NM_025349 | lsm7 homolog, U6 small nuclear RNA associated (*S. cerevisiae*) | 1.5 | NM_009279 | signal sequence receptor, delta Ssr4/ Trap | 1.8 |
| NM_133195 | Bruno-like 4, RNA binding protein (*Drosophila*) | 2.4 | NM_010926 | neighbor of Cox4 | 2.1 |
|  |  |  | NM_027121 | vitamin K epoxide reductase complex, subunit 1-like 1 | 1.7 |
|  | **Survival/anti-apoptosis** |  | NM_009729 | ATPase, H+ transporting, V0 subunit C | 1.9 |
| NM_026669 | bax-inhibitor-1 | 1.6 | NM_144870 | similar to NADH dehydrogenase (ubiquinone) Fe-S protein 8 | 1.8 |
| NM_010015 | defender against cell death 1 | 1.8 | NM_025313 | ATP synthase delta chain, mitochondrial precursor | 2.2 |
| NM_009689 | survivin | 2.1 |  |  |  |
|  |  |  |  | **Nucleotide metabolism** |  |
|  | **Kinase/phosphatase** |  | NM_018737 | cytidine 5'-triphosphate synthase 2 | 1.6 |
| NM_008974 | protein tyrosine phosphatase 4a2 | 1.8 | NM_013556 | hypoxanthine guanine phosphoribosyl transferase | 1.9 |
| NM_023138 | mitogen activated protein kinase kinase 2, MEK2 | 1.5 |  |  |  |
| NM_009594 | v-abl Abelson murine leukemia oncogene 1/ c-Abl | 1.8 |  | **Unknown function** |  |
| NM_032418 | dystrophia myotonica kinase, B15 | 1.5 | NM_011712 | ww domain binding protein 5 | 1.5 |
|  |  |  | H3125H02 | unknown | 1.5 |
|  | **Signal transduction** |  | H3044G08 | unknown | 1.9 |
| NM_009919 | cornichon homolog (*Drosophila*) | 1.9 | NM_177730 | RIKEN cDNA 1110001C20 gene | 1.5 |
| NM_028303 | similar to *Drosophila* Discs large-1 tumor suppressor protein | 1.8 | NM_199467 | hypothetical protein F730047E07 | 1.5 |
| NM_009007 | rac1 | 1.6 | NM_026681 | hypothetical protein 0610010D24 | 1.5 |
|  |  |  | XM_204062 | RIKEN cDNA A630031M04 gene | 1.9 |
|  | **Transcription** |  | H3026B09 | unknown | 1.6 |
| NM_022309 | core binding factor beta/ PEBP2 beta | 1.6 | BQ044654 | RIKEN cDNA 2700094K13 gene | 1.8 |
| NM_007681 | centromere autoantigen A | 1.7 |  |  |  |
| NM_025652 | general transcription factor III A (gtf3a) | 2.3 |  |  |  |
| XM_149258 | zinc finger protein 217 | 1.8 |  |  |  |
